# Beyond Hesitancy: Current Multi-Level Polio Vaccine Refusals and Pathways to Acceptance in a High-Risk Region of Pakistan

**DOI:** 10.3390/vaccines14080719

**Published:** 2026-08-20

**Authors:** Farhana Tabassum, Maha Azhar, Amal Khalid, Mushtaque Mirani, Narjis Fatima Hussain, Sujeet Lohana, Aadarsh Fateh Muhammad, Khadija Ali, Jai K Das

**Affiliations:** 1 Institute for Global Health and Development, The Aga Khan University, Karachi 74800, Pakistan; farhana.tabassum@aku.edu (F.T.); maha.azhar22@alumni.aku.edu (M.A.); amal.khalid@aku.edu (A.K.); mushtaque.mirani@aku.edu (M.M.); sujeet.lohana@aku.edu (S.L.); aadarsh.fateh@aku.edu (A.F.M.); khadijasyeda224@gmail.com (K.A.); 2 Department of Nutritional Sciences, Faculty of Medicine, Medical Sciences Building, University of Toronto, Toronto, ON M5S 1A8, Canada; narjisfatima.hussain@mail.utoronto.ca; 3 Centre for Global Child Health Hospital for Sick Children, Toronto, ON M5G 1X8, Canada; 4 Department of Paediatrics and Child Health, The Aga Khan University, Karachi 74800, Pakistan

**Keywords:** polio, vaccine refusal, vaccine hesitancy, Pakistan, operational and communication guideline

## Abstract

Background: Pakistan is one of only two countries where wild poliovirus type 1 (WPV1) continues to persist. Vaccine refusals challenge efforts to end the virus, especially in high-risk communities. Methods: A qualitative exploratory study took place from September to November 2025 in three High-Risk Union Councils (HRUCs) of Karachi, Pakistan. The study examined reasons for refusing the polio vaccine and identified specific communication and operational strategies to improve vaccine acceptance. A total of 23 in-depth interviews and 10 focus group discussions were conducted with 71 participants, including parents who refuse the vaccine, polio program staff, physicians, and government stakeholders. Data were analyzed using a sequential inductive and deductive thematic approach, mapping findings onto the Socio-Ecological Model (SEM) with NVivo 15 (Lumivero, Denver, CO, USA). Results: Factors influencing polio vaccine refusals were interconnected and spanned multiple levels. At the intrapersonal level, campaign fatigue, a lack of knowledge about poliomyelitis, concerns about side effects, and rumors about infertility were common. Community-level factors included household gatekeepers, local influencers, and digital misinformation. Institutional barriers included weak communication from the frontline, inadequate supervision, workforce shortages, poor tracking of refusals, and limited involvement of physicians. At the policy level, refusals were linked to distrust in governance, poor municipal services, and dissatisfaction with vertical campaign methods. Recommended strategies included integrating polio services with routine immunization and primary healthcare, strengthening counseling by physicians, engaging trusted local influencers and community leaders, expanding targeted social media campaigns to counter misinformation, implementing quick responses to rumors, enhancing workforce capacity, and using data-driven microplanning, including targeted SNIDs and focused interventions in areas with chronic refusals. Conclusions: Polio vaccine refusals are shaped by social, communication, operational, and structural factors. Improving communication and operational guidelines by reducing the number of campaigns, increasing active engagement at the frontline, integrating polio activities into routine health services, and addressing misinformation with a dynamic communication strategy may enhance vaccine acceptance and support polio eradication efforts.

## 1. Introduction

Immunization is a crucial public health practice that prevents disease and disability, promotes healthy growth and lifestyles, and saves 3.5 to 5 million lives each year [1,2]. Vaccination programs help improve life expectancy and support health equity, especially in lower- and middle-income countries facing major infectious diseases [3]. The World Health Organization (WHO) recognizes immunization programs as fundamental to global public health and essential for universal health coverage [4].

Poliomyelitis has been a significant health concern and a key focus of global eradication efforts among vaccine-preventable diseases [5,6]. The Global Polio Eradication Initiative (GPEI), launched in 1988, reduced global polio incidence by over 99%, dropping from around 350,000 cases per year in more than 125 endemic countries to a small number of cases in specific areas of Pakistan and Afghanistan. In these countries, WPV1 transmission remains endemic and poses a serious risk to global eradication efforts, indicating the potential for international spread [7,8].

In 1978, Pakistan established the Expanded Programme on Immunization (EPI) to provide routine protection against major childhood diseases, including tuberculosis, diphtheria, pertussis, tetanus, measles, and poliomyelitis [9]. This initiative highlighted the need for an intensified campaign against polio, leading to the launch of a dedicated Polio Eradication Programme in 1994 with support from national and international partners. Since then, a comprehensive eradication infrastructure has developed, including supplementary immunization activities, National and sub-National Immunization Days, and environmental surveillance systems through Emergency Operations Centers (EOC) at federal and provincial levels [9]. These coordinated efforts have significantly reduced the number of paralytic polio cases and enhanced outbreak detection and response capabilities [9].

Despite this progress, the final phase of polio eradication is increasingly challenging [10]. National case counts have declined, but persistent transmission continues in a limited number of high-risk areas marked by overcrowding, population mobility, poor sanitation, low routine immunization rates, and groups of unvaccinated children [11,12]. In Pakistan, many vaccine refusals and poliovirus detections are concentrated in certain High-Risk Union Councils (HRUCs), particularly in Karachi and some districts of Khyber Pakhtunkhwa (KP), which remain major reservoirs for virus transmission. These areas consistently report vaccine refusals, missed children, and the detection of poliovirus, underscoring the need for tailored operational and communication strategies [13,14,15].

Recent evidence indicates that the remaining barriers to polio eradication are more about communication and operational challenges than just biomedical issues. Misinformation, fear of side effects, distrust of institutions, household decision-making dynamics, campaign fatigue, and dissatisfaction with public services all contribute to these barriers [16,17].

**Figure 1:**
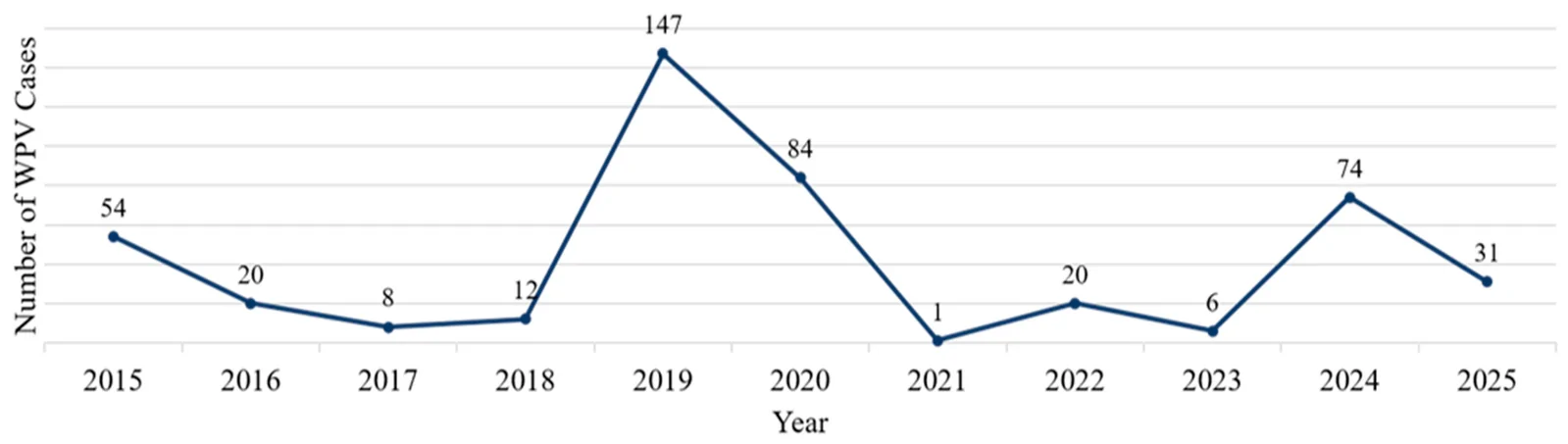
WPV Case Count in Pakistan [18].

While past research has looked at vaccine hesitancy in general populations, this study focuses on high-risk refusal regions. It explores how social, communication, and operational elements interact at various levels to design context-specific guidelines that can enhance trust, improve program performance, and increase vaccine uptake (Figure 1).

## 2. Materials and Methods

### 2.1. Study Design

A qualitative exploratory study was carried out from September to November 2025 to identify sociocultural, religious, and systemic factors that contribute to polio vaccine refusal. The study also aimed to explore and refine communication and operational guidelines to encourage vaccine acceptance in areas prone to refusals.

### 2.2. Theoretical Framework

**Figure 2:**
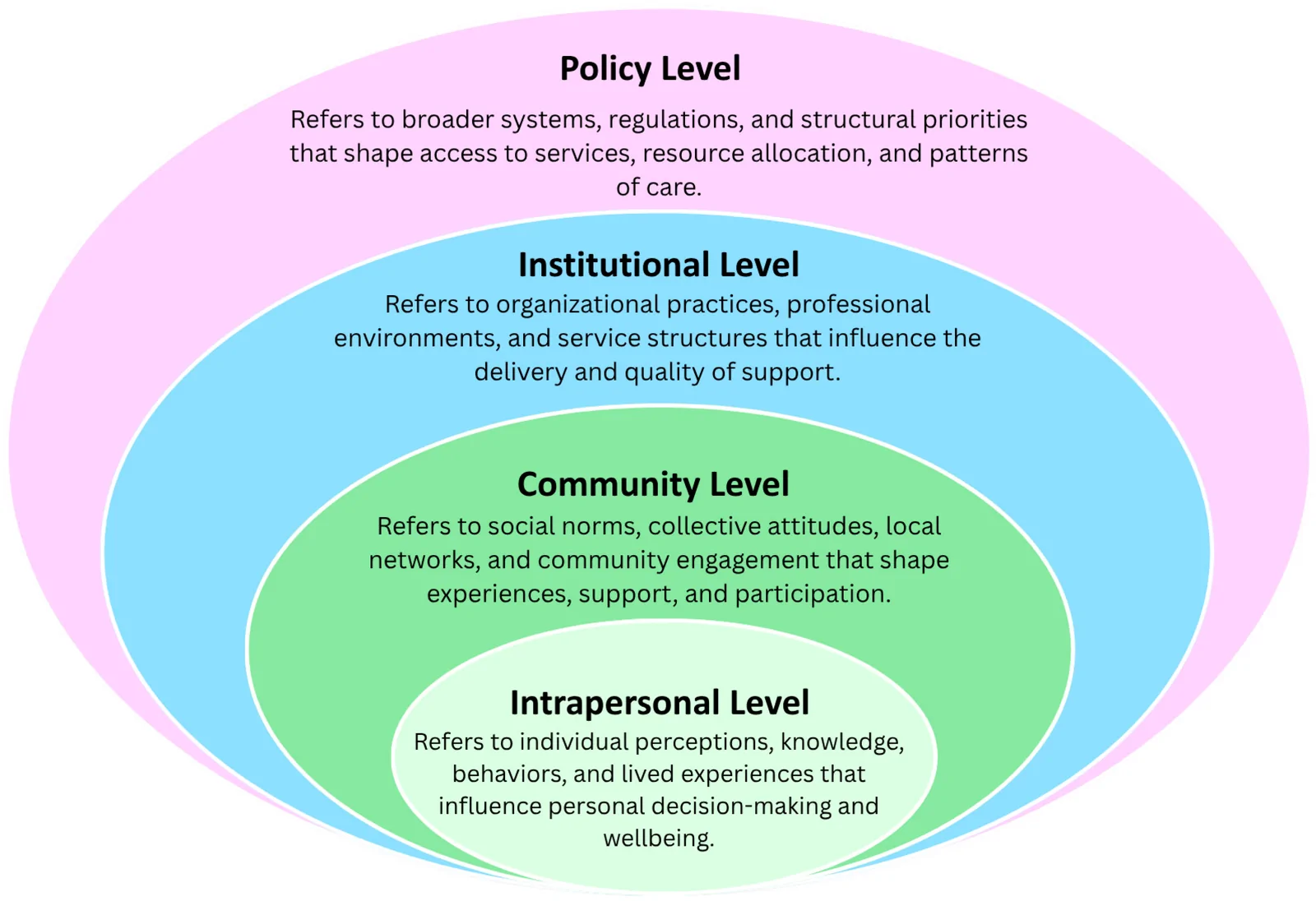
Socio-Ecological Model [21,22].

This study used the Socio-Ecological Model (SEM), which recognizes that health behaviors are influenced by multiple, interacting factors at interpersonal, community, institutional, and policy levels [19]. The SEM was chosen because polio vaccine refusal is complex and shaped by social relationships, community norms, health system experiences, and broader governance contexts [19,20,21] (Figure 2).

At the intrapersonal level, the model examines factors like knowledge, risk perceptions, beliefs about vaccine safety, and past vaccination experiences. At the community level, family decision-making dynamics, social networks, religious influences, and local norms can affect views on polio vaccination. The institutional level focuses on interactions with health workers, service quality, program implementation, and trust in healthcare systems. Lastly, the policy level looks at larger structural influences, such as governance, public services, and policies that affect community trust and vaccine acceptance.

### 2.3. Study Setting

The study was conducted in three HRUCs of Karachi, Pakistan. These areas were selected in consultation with the EOC, a collaborative body responsible for coordinating polio eradication efforts. Selection was based on program surveillance data and EOC risk classifications, identifying these HRUCs as ongoing hotspots for vaccine refusals, representing about 31% of Karachi’s recent refusals. Additionally, these areas consistently show active poliovirus circulation through reports of acute flaccid paralysis and environmental (wastewater) surveillance, signaling a continued risk of transmission. EOC involvement ensured that the selection of study sites relied on data evidence and remained relevant to local contexts and operational priorities.

### 2.4. Participants and Sampling

This study used purposive sampling to recruit participants for in-depth interviews (IDIs) and focus group discussions (FGDs) from the selected union councils [23]. This method allowed researchers to select individuals directly involved in or affected by polio immunization efforts, capturing diverse views on vaccine acceptance, including parents with a history of refusing the polio vaccine.

Snowball sampling was also used to find key informants with expertise or influence in vaccination efforts, such as government officials, general physicians, polio workers, and community mobilizers [24]. All participants met specific eligibility criteria to ensure data relevance, consistency, and quality (Table 1).

### 2.5. Interview Guide

Semi-structured interview guides were developed for IDIs and FGDs to collect role-specific experiences. The development process included a thorough literature review on vaccine hesitancy, followed by multiple critical reviews by the research team and public health experts. This iterative process helped ensure that questions were open-ended and relevant to parents, polio program staff, physicians, and government stakeholders. The guide included flexible probes allowing participants to elaborate on important subjects while maintaining consistency across interviews. The semi-structured format provided enough structure for comparability while enabling deeper exploration of emerging topics. The interview guides were translated into Urdu and reviewed by experts for clarity and relevance before use, with continuous refinements based on field experience. A summary of the key areas of enquiry for each stakeholder group is presented in Table 2.

### 2.6. Data Collection

Data were gathered through face-to-face IDIs and FGDs conducted by sociologists trained in public health qualitative research, using semi-structured guides. This approach ensured consistency while allowing participants to share their views and experiences using their own words. Data collection continued until saturation was reached, with each IDI and FGD lasting about 35 to 50 min [25]. Interviews and discussions were held at participants’ homes or workplaces based on their preferences for privacy, comfort, and ease of participation. Prior to data collection, written informed consent was obtained to collect data and use it further for publication purposes.

With participants’ permission, interviews were audio recorded using digital voice recorders to capture responses accurately. All interviews were conducted in Urdu, and we later transcribed and translated the audio recordings into English for analysis.

### 2.7. Data Analysis

Data were analyzed using a sequential inductive and deductive thematic approach. Initially, transcripts were read multiple times to achieve data familiarization, and open coding was applied to capture participants’ language, meanings, and experiences related to polio vaccine refusal [26]. NVivo 15 (Lumivero, Denver, CO, USA) was used to organize, manage, and code the data, facilitating the systematic identification and exploration of themes. Subsequently, a deductive analysis was conducted using the Socio-Ecological Model (SEM) as an analytical framework. Emergent themes were mapped across the intrapersonal, community, institutional, and policy levels to examine how factors influencing polio vaccine refusal interacted across multiple levels. To ensure analytical rigor, themes were iteratively reviewed and discussed between FT and MA, while triangulation across diverse participant groups and data sources was used to enhance the credibility and validity of the findings.

## 3. Results

We conducted twenty-three IDIs and ten FGDs with 71 participants (Table 3). The average age of FGD participants was 32.78 ± 9.09 years, while the average age for IDIs was 37 ± 11.51 years. Participants included housewives (39.13%), government officials (30.42%), medical professionals (13.04%), and others engaged in manual or private work. Educational backgrounds varied, with representation from primary to higher education levels. Participants spoke multiple languages, mainly Pashto, Urdu, Hindko, and Balochi, reflecting the study population’s linguistic diversity.

### 3.1. Intrapersonal Level

At the intrapersonal level, parents who refused the polio vaccine often lacked a clear understanding of poliomyelitis, how it spreads, the need for multiple oral doses, the age eligibility for vaccination, and the serious consequences of paralysis. Specific misinformation topics also caused refusals including persistent rumors about the vaccine containing harmful ingredients (e.g., animal-derived substances), causing future infertility, or fear of acute side effects like fever or diarrhea. These beliefs were frequently amplified by digital platforms like TikTok and WhatsApp, which spread negative content rapidly before official campaign communications could respond. Many parents noted that polio program staff had not provided strong explanations for the need for repeated doses, leading them to believe that frequent campaigns were pointless or unnecessary. Frontline workers also shared this frustration, stating they often lacked the time or resources to provide detailed answers, making it difficult to address complex questions from parents. Additionally, several parents distinguished between routine immunizations and supplementary polio doses, often trusting injectable vaccines given in hospitals more than oral drops provided at home.

Fear of side effects was another significant concern for parents. They viewed fever, diarrhea, weakness, or any illness following vaccination regardless of any causal connection as harmful. Many reported a lack of follow-up or support when such negative effects occurred. Persistent beliefs about infertility also emerged among refusal parents, who linked repeated doses to future fertility issues or foreign agendas. These beliefs were often fueled by rumors within their social circles and on digital platforms.

Participants expressed physical and psychological fatigue due to the frequent vaccination campaigns, describing repeated visits every few weeks and the resulting frustration associated with recurring discussions with polio workers, and frontline workers expressed similar exhaustion. They described the repetitive nature of campaigns as a barrier to forming meaningful connections with families. Many parents felt upset and neglected, believing their children’s health needs were being overshadowed by the singular focus on polio. Relevant excerpts from this level and others are presented in Table 4.

#### 3.1.1. Communication Guideline

Participants suggested that messages should shift from general awareness to brief, frequent counseling that emphasizes polio transmission, the necessity for repeated doses, and the fact that paralysis has no cure, specifically targeted to high-risk areas. To dispel myths, they recommended implementing a “Responsive Information System” where physicians and community leaders are trained to address specific rumors about infertility and side effects through localized social media content and face-to-face discussions outside of polio campaigns. The role of pediatricians and general physicians was deemed essential, as parents typically trust them regarding their children’s health.

#### 3.1.2. Operational Guideline

Participants, especially government stakeholders, recommended that frontline teams should bring simple counseling cards, side-effect referral slips, and lists of nearby EPI/fixed centers, so that participants are adequately referred. They also suggested conducting follow-up visits for households that reported adverse reactions or ongoing concerns. Furthermore, there was strong agreement on the need to formalize a “Referral and Support Protocol” so households reporting issues would receive prompt follow-up from a trained health official instead of just a volunteer. To reconcile the repetitive nature of campaigns with the need for information, the program should streamline door-to-door vaccination visits to reduce fatigue, while focusing targeted, high-quality counseling through various general channels, exclusively on addressing information gaps and myths, ensuring engagement is meaningful rather than intrusive.

### 3.2. Community Level

Participants noted that female household members often lacked the freedom to make decisions about vaccination; their choices were heavily influenced by fathers, male elders, relatives, and neighborhood leaders. Mothers typically wanted their children vaccinated but were often overruled by husbands or senior male family members. In some communities, tribal, linguistic, and kinship identities played a significant role in trust, with residents favoring communicators from their own ethnic or linguistic backgrounds.

Respondents consistently mentioned that local doctors, mosque leaders, elected officials, and respected elders had greater persuasive power than temporary campaign workers. However, many participants noted that these influential figures were underutilized by the program. Religious leaders had mixed influences; they were helpful in certain areas but fueled doubt in others. Digital media was also recognized as a powerful force; government stakeholders and field staff observed that negative content on TikTok, Facebook, and WhatsApp spread swiftly, shaping public perception more effectively than official messages.

#### 3.2.1. Communication Guideline

Participants suggested using joint Friday sermons led by physicians, religious leaders, and polio communication officers to address myths and concerns among fathers and other male family decision-makers. They also emphasized the importance of responding quickly to rumors circulating on digital platforms with accurate, culturally appropriate information. Involving trusted local digital influencers including popular TikTok creators from the predominant linguistic and ethnic groups within the HRUCs was seen as a valuable way to make vaccination messages more relatable and credible within the community.

#### 3.2.2. Operational Guideline

Participants emphasized that program managers should conduct influencer mapping to identify chronic refusal areas and engage local negotiators, to lead refusal-conversion teams instead of solely relying on external staff. Key influencers in areas with high refusal rates should be identified and actively involved.

### 3.3. Institutional Level

Participants often pointed to institutional barriers as significant problems, specifically weaknesses in recruiting, training, supervising, and holding frontline teams accountable. While local recruitment was crucial to address cultural and linguistic needs, newly hired workers frequently lacked confidence, technical knowledge, and communication skills. Government stakeholders described the training process as being too theoretical, rushed, and lacking practical field simulations, leaving workers unprepared for real-world interactions.

Frontline staff remarked that heavy workloads and constant pressure to meet rapid coverage targets reduced opportunities for meaningful engagement with households. These pressures compromised data quality; inadequate monitoring and poor evening review meetings led to incorrect recording of refusals and misclassification of missed children. While officials noted that data were collected, it was rarely used for targeted programming, limiting the program’s ability to respond to challenges in the field.

Community confidence was further damaged by some teams’ behaviors. Parents criticized workers for being argumentative, threatening, or hurried during interactions. Also, the lack of visible identification, poor hygiene practices, and concerns about how vaccines were handled eroded parental trust. Lastly, there was a significant gap in involving healthcare providers. Most refusal parents noted that their physicians hardly ever discussed polio vaccination during clinic visits. Officials stressed that a brief, credible recommendation from a physician would be far more effective than repeated household pressure; however, public health facilities were seldom integrated into polio eradication efforts, and were often overwhelmed and under-resourced, further diminishing community trust in the overall health system.

#### 3.3.1. Communication Guideline

Participants consistently highlighted the need to shift frontline staff training from theoretical manuals to competency-based simulation training. This should include role-playing specific scenarios, such as empathetic refusal conversion using active listening, managing rumors with evidence-based rebuttals, and addressing parental concerns regarding vaccine ingredients and cold-chain integrity. Training programs must ensure that teams are equipped to respond confidently and respectfully to challenging questions from community members during household visits. Addressing vaccine refusal in a fair and truthful manner requires shifting from coercive enforcement to informed consent based on trust. This involves clear, empathetic dialog where frontline workers explain the necessity of multiple doses and address safety concerns, ensuring that parents are fully informed partners in their children’s health decisions rather than targets of pressure.

#### 3.3.2. Operational Guideline

Reinforce merit-based recruitment, offer practical training, provide supportive supervision, track refusals, and use campaign data daily. Visible identification cards and uniforms should be mandatory to ensure professional recognition. Hygiene standards must be strictly upheld, requiring frontline workers to sanitize hands before entering each home, maintain clean and tidy attire, and wear personal protective equipment (PPE) as needed to demonstrate respect for household health and safety. Moreover, standardize cold-chain demonstrations, such as showing the vaccine vial monitor (VVM) status to parents, to visually assure them of vaccine quality. Establishing clinic-based “Polio Counseling Desks” was another suggestion, where physicians could reinforce campaign messages and pediatric academic bodies should be included. Operational reforms should focus on workforce quality and establish realistic performance expectations that prioritize meaningful engagement with families rather than rapid campaign completion. The systems for data integrity and refusal tracking should be strengthened to enable more targeted responses. In addition, polio eradication activities should be formally integrated with routine immunization services and health facilities as a standard component of service delivery.

### 3.4. Policy Level

At the policy level the issue of vaccine refusal was deeply embedded within a larger context of distrust towards institutions and dissatisfaction with municipal services delivery. Participants noted that the state only communicates during polio campaigns, thus causing discontent among the deprived populations. The refusal parents and government officials highlighted that there is a list of unmet demands such as unreliable supply of clean drinking water, sanitation, electricity, infrastructure, and access to medicines which caused feelings of marginalization of the communities. It created the impression that the state does not have the right priorities, spending its resources on frequent visits for polio vaccinations and ignoring the urgent needs of the people.

In addition, the participants criticized the vertical structure of the polio program, which works in silos and is not integrated with routine immunization, maternal and neonatal child health (MNCH), nutrition and social protection services. It not only decreases the sustainability of the program but also prevents building community ownership.

Stakeholders expressed differing views on enforcement policies. While some officials supported linking vaccination status to national identity cards, school admission, SIM card registration, or other sanctions, others cautioned against such measures. They emphasized that coercive approaches may foster resentment within communities, encourage superficial or false compliance, and further erode trust in frontline health workers.

#### 3.4.1. Communication Guideline

Awareness sessions should be conducted through existing community platforms, including waiting areas of health facilities, union council meetings and community town halls. These sessions should be facilitated by trusted local voices, particularly social protection workers, elected representatives, who can frame immunization as a matter of child well-being, dignity, and health rights. Integrating vaccine messages with information on routine health and social services may further strengthen community trust and engagement.

#### 3.4.2. Operational Guideline

To address the overfocus on polio, it was proposed to strengthen interdepartmental coordination (e.g., with sanitation and utility departments) as a standard planning and execution issue. Integrating polio eradication activities with routine immunization, maternal and child health (MNCH), and social protection services is essential to demonstrate government commitment to overall child well-being, effectively moving beyond a siloed focus on polio. The polio program needs to shift from its vertical structure to an integrated service delivery approach, aligned with the EPI, MNCH, nutrition, and social protection services. In order to respond to the problem of community frustration, the polio program needs to coordinate pre-campaign municipal-level activities such as waste disposal and clearance of drainage channels in addition to ensuring reliable clinic timings and provision of medicines. The accountability mechanism should not be based on enforcing rules but on creating incentive mechanisms where the team with high community engagement is rewarded. Lastly, an “Early Warning System” for rumor detection needs to be institutionalized at the district and UC levels which will allow responding rapidly and locally, keeping service quality and community response in mind over the pace of campaigns.

### 3.5. Stakeholder Validation of Communication and Operational Guidelines

At the completion of the study, the research results along with the proposed communication and operational guidelines were discussed during dissemination meetings with Provincial Emergency Operations Centre (EOC) in February 2026 and with the National Emergency Operations Centre (NEOC) in March 2026. The proposed recommendations were well supported by the stakeholders, who emphasized the need for more involvement of physicians, communication skills training for the workforce, social media responses to counter fake information, better monitoring and data systems and finally integration of polio activities with other services such as routine immunizations, maternal and child health, nutrition and social protection services. These discussions supported the feasibility and programmatic relevance of the proposed recommendations.

## 4. Discussion

This study explored the multi-level determinants of polio vaccine refusal in high-risk urban communities of Karachi using the Socio-Ecological Model. The findings demonstrate that vaccine refusal is shaped by interconnected influences operating at the intrapersonal, community, institutional, and policy levels rather than by simple opposition to vaccination. Refusals were commonly associated with limited understanding of poliomyelitis, concerns about side effects and infertility, campaign fatigue, the influence of household gatekeepers and community norms, digital misinformation, weaknesses in frontline communication and program implementation, and broader dissatisfaction with public services and government responsiveness. Many refusals were conditional and could shift when families received respectful engagement, credible information, and support from trusted messengers. Illustrative quotations supporting these findings are presented in Table 4.

**Figure 3:**
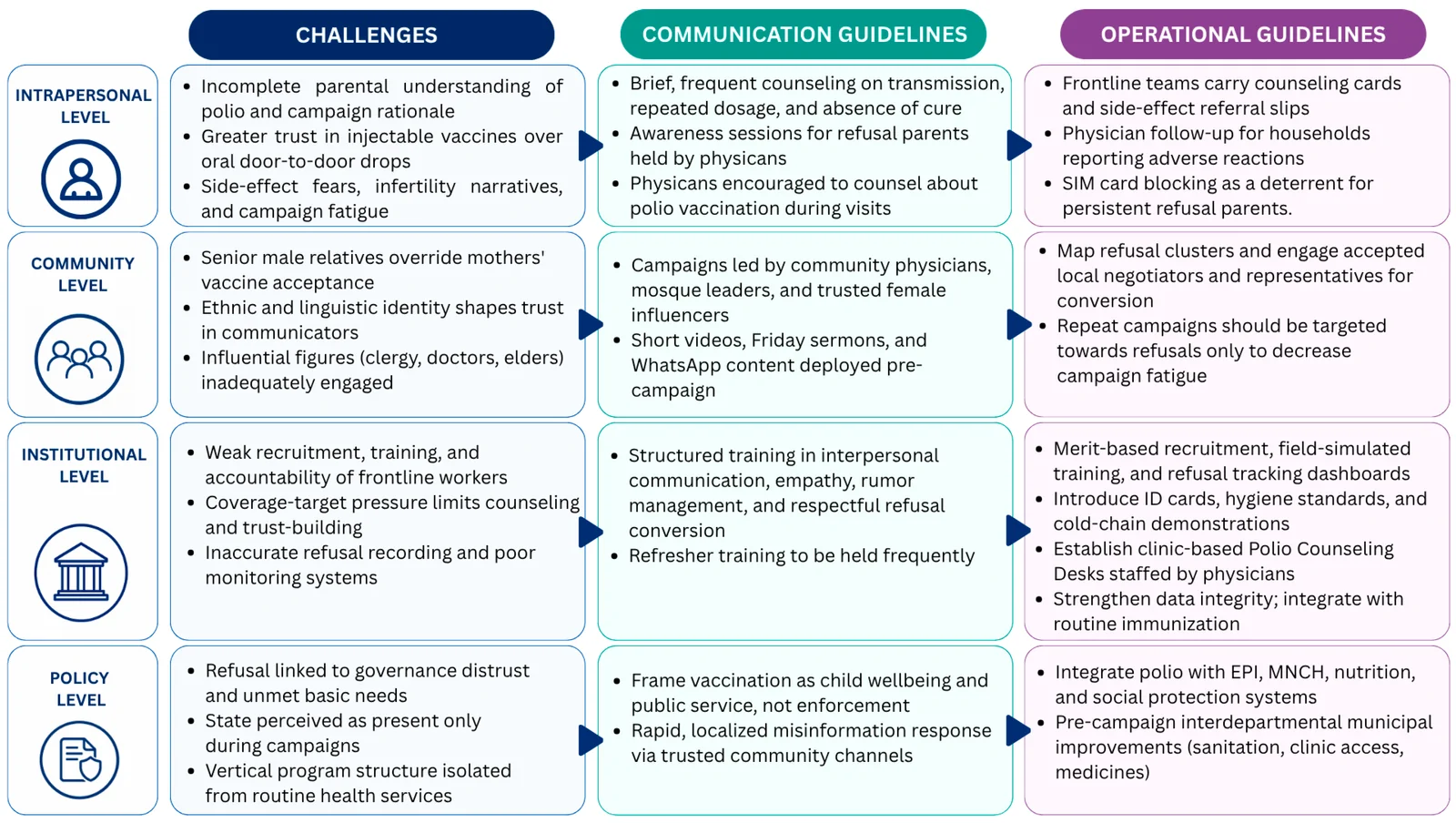
Communication and operation guidelines.

The study also identified practical, context-specific communication and operational strategies that may strengthen vaccine acceptance and support the final stages of polio eradication in these high-risk settings (Figure 3).

At the intrapersonal level, parents mentioned their limited understanding of polio, doubts about needing repeated oral doses, concerns about side effects, rumors about infertility, and fatigue from ongoing campaigns [17,27,28]. Frontline workers also described similar feelings of fatigue and a lack of chances to answer parents’ questions adequately. Parents expressed more confidence in injectable vaccines given at healthcare facilities compared to door-to-door oral polio vaccine and pointed out the absence of follow-up after reports of adverse events [29,30,31]. These findings highlight the need for stronger communication, structured referral mechanisms, and closer integration of polio services with routine healthcare [14].

On the community level, the decisions around child vaccination were heavily influenced by fathers, elders and influential relatives [15,32]. Trust was also shaped by shared language, ethnicity, and neighborhood identity, with local physicians, respected elders, religious leaders, and community influencers considered more credible than temporary campaign workers [14,15,32,33]. Participants emphasized the need to recruit community mobilizers who speak local languages such as Pashto, Balochi, Sindhi, and Urdu to improve rapport and facilitate refusal conversion. Evidence also suggests that locally recruited mobilizers who share the community’s language and cultural background are more effective in addressing vaccine hesitancy and improving acceptance [15,34].

Digital misinformation emerged as another major driver of vaccine refusal [35]. Participants consistently identified TikTok, WhatsApp, Facebook, and informal community networks as major sources of misinformation, mainly regarding the vaccine causing infertility and untrustworthy vaccine contents [35,36]. Frontline workers pointed out that false narratives about polio vaccination often spread quickly before campaigns begin in Pakistan, making it challenging for official messages to reach communities in time [35]. Respondents therefore recommended rapid, locally tailored responses to misinformation led by trusted physicians, community influencers, religious leaders, and respected public figures. These findings suggest that communication strategies should move beyond one-way awareness campaigns and toward proactive rumor monitoring and culturally sensitive digital engagement that addresses concerns as they arise [15,37]. These findings reflect growing evidence that misinformation shared through digital media is reducing confidence in vaccines worldwide [36,38,39].

Institutional barriers also played a substantial role in sustaining vaccine refusals. Weak recruitment systems, inadequate practical training, limited supervision, excessive workloads, poor interpersonal communication, and inaccurate refusal tracking reduced opportunities for meaningful engagement with families. Refusal is influenced not only by community perceptions but also by program performance and service quality [40,41]. Another important finding was the limited involvement of physicians despite their high level of community trust. Participants proposed competency-based simulation training, clinic-based polio counseling desks, visible staff identification, standardized cold-chain demonstrations, and strengthened data systems as practical operational improvements to enhance program credibility [41,42].

At the policy level, participants associated vaccine refusal with broader dissatisfaction regarding public services and government responsiveness. Communities questioned why repeated vaccination campaigns continued while basic needs such as water, sanitation, electricity, healthcare, and infrastructure remained inadequately addressed. Although some stakeholders supported coercive measures, participants generally believed that enforcement through police involvement or service restrictions weakened trust and encouraged superficial compliance rather than genuine acceptance [42]. These findings support greater integration of polio activities with routine immunization, maternal and child health, nutrition, and social protection services to strengthen community trust and program sustainability.

## 5. Conclusions

The persistence of polio vaccine refusal in high-risk urban communities reflects not only concerns about vaccination itself but also broader issues of trust, communication, service delivery, and governance. Sustainable progress toward eradication will require shifting from repetitive mass campaigns to integrated, people-centered approaches that prioritize respectful engagement, trusted local voices, responsive communication, and strong frontline systems. Embedding polio activities within routine primary health care and using data-driven, community-responsive strategies may be critical to building lasting vaccine confidence and achieving a polio-free Pakistan.

## Figures and Tables

**Table 1:** Sampling frame for focus group discussions and in-depth interviews.

Stakeholder Category	Granularity	Frequency	Inclusion Criteria
**Focus Group Discussion**			
Government stakeholders	Outbreak response team (OBR), district focal persons, Deputy district polio officer (DDPO) immunization officers, WHO representatives and Director of General Health	02	Directly or indirectly involved in designing, planning, coordination, and implementation of polio eradication activities.
Polio program staff	Polio workers	05	Minimum 5 years of experience in door-to-door polio vaccination campaigns and outreach.
	Communications officers	03	Minimum 5 years of experience in health communication, social mobilization, and community engagement within polio eradication programs.
**Total**		**10**	
**In-depth Interviews**			
Parents	Refusal Mothers	09	Parents with a history of refusing polio vaccination for their children during polio immunization campaign.
	Refusal Fathers	09	
Government Stakeholders	Ex-DG Health Sindh	01	Directly and indirectly involved in planning, coordination, monitoring, and implementation of public health or administrative activities related to childhood vaccination and polio campaign.
	Assistant Commissioner	01	
	Additional Deputy Commissioner	01	
Renowned General Physicians	From Baldia Town	02	Recognized as a trusted physician in the targeted community, with involvement in public health, primary care, and immunization services.
**Total**		**23**	

**Table 2:** Key thematic domains from the interview guide.

Refusal Parents	Frontline Workers	Government Stakeholders
Themes		
Perceptions of polio and vaccines Community practices Role of healthcare providers in polio awareness Trust in health services Counseling sessions by the polio program Misinformation and myths Vulnerabilities and barriers Experiences with missed opportunities Suggestions for improvements	Community attitudes, perception and challenges Polio vaccination access Managing refusals and community interactions Community awareness activities Role of healthcare providers in polio awareness Vulnerability, barriers and resources Community resources and coping strategies Suggestions for improvement	Current polio and immunization policies Government role and coordination Trust, community engagement and communication Barriers and systemic challenges Future directions and policy recommendations

**Table 3:** Demographic indicators.

Indicators	IDIs (*n* = 23)	FGDs (*n* = 71)
**Age (years), Mean ± SD**	37.0 ± 11.51	32.78 ± 9.09
**Mother Education (*n* = 9)**		
Illiterate	5 (55.6%)	—
Primary Education	—	—
Middle Education	3 (33.3%)	—
Secondary Education	1 (11.1%)	—
**Father Education (*n* = 9)**		
Illiterate	1 (11.1%)	—
Primary Education	1 (11.1%)	—
Middle Education	2 (22.2%)	—
Secondary Education	1 (11.1%)	—
Higher Secondary Education	2 (22.2%)	—
Higher Education	2 (22.2%)	—
**Government Stakeholder Education (*n* = 5)**		
Secondary Education	—	28 (39.44%)
Higher Secondary Education	—	27 (38.03%)
Higher Education	5 (100%)	16 (22.54%)
**Occupation**		
Polio Worker	—	41 (57.75%)
Community Mobilizer	—	21 (29.58%)
Technical Expert	—	9 (12.67%)
Manual/Fieldwork	2 (8.69%)	—
Medical Profession	3 (13.04%)	—
Housewife	9 (39.13%)	—
Government Official	7 (30.43%)	—
Service/Private	2 (8.69%)	—
**Primary Language**		
Urdu	—	20 (28.17%)
Pashto	12 (52.17%)	35 (49.30%)
Hindko	5 (21.74%)	16 (22.54%)
Balochi	6 (26.09%)	—

**Table 4:** Selected interview excerpts categorized by themes emerging from the SES analysis.

SEM Level	Theme	Representative Quote
**Intrapersonal**	Limited knowledge of polio virus and immunization	“*I do not know of any vaccination center, nor have I been informed about it. Instead of force-feeding the vaccination at our doorsteps, we should be informed about the necessity of the vaccine.*” (Refusal Mother) “*We would prefer going to the hospital for the polio vaccine like we do for the other vaccines*.” (Refusal Mother) “*Parents’ awareness in terms of severity, treatment, how many doses are needed, and until what age they should be given is very limited*.” (Government Stakeholder)
	Fatigue from repeated campaigns and visits	“Frequent visits every 15 days, especially accompanied by police, are very troubling and increase resistance.” (Refusal Father) “*Children are continuously subjected to repeated vaccinations. Every few months there is another campaign, another vaccine, and people are getting tired of it*.” (Government Stakeholder)
	Fear and misconceptions	“*I’ve seen on videos stating that the vaccine is harmful, causes infertility, and contains bad medicines or animal urine. Our neighbors say that the vaccine also causes poliovirus*.” (Refusal Father) “*The first question they ask is about the losses and harms they have heard about. Negative propaganda spreads much faster than factual information*.” (Physician)
**Community**	Influence of ethnicity, language, and social identity	“*If there is a person from their own community and their own language, they will be able to counsel them much better*.” (Frontline Worker)
	Influence of social media and trusted figures	“*We trust our neighborhood leaders and religious leaders. If they doubt the vaccine, word about its authenticity spreads fast*.” (Father Refusal) “*People from all backgrounds, ethnicities and class are on social media. We should use this platform to spread awareness*.” (Frontline Worker)
	Community demands and competing priorities	“*They (parents) say, look at the garbage here, look at the sewage problems. Instead of talking about polio, talk about these issues*.” (Frontline Worker)
	Household Authority and Polio Vaccination Decisions	“*I typically wanted to give polio drops to my child but often overruled by husbands or senior male family members*.”
**Institutional**	Lack of physician engagement	“*Doctors never ask about the polio vaccine during checkups. They only ask about routine vaccines. That’s why we trust routine vaccines but not polio drops. If the doctors encouraged us my family would easily agree*.” (Refusal Mother) “*We have been working on polio eradication for so many years, but we could not make the same difference that doctors make*.” (Government Stakeholder)
	Weak frontline training and preparedness	“*These polio workers don’t know enough themselves. When we ask questions, they have no answers. They just say videos are fake with no explanation. Most have not been trained on the virus*.” (Refusal Father) “*Sometimes they do not even complete the training and suddenly come into the field. They do not know how to carry the vaccine or answer questions*.” (Physician)
	Pressure-driven implementation	“*I can assure you, no matter how much stamina or energy someone has, no single worker can visit 20 refusal households in a day. This pressure leads to fake entries. This is why we need to plan refusal conversion strategies across influencers and representatives*.” (Frontline Workers) “*Workers are pressured that if they don’t come back after administering polio drops, their job will be terminated*.” (Government Stakeholder)
**Policy**	Vertical nature of the program	“*The program is going on vertically, in a separate silo, and people are struggling at the field level*.” (Government Stakeholder)
	Governance and service delivery concerns	“*Why is the government only coming for polio? People ask about water, sanitation, roads, and other problems that remain unresolved. Children have other diseases too that no one helps for*.” (Refusal Father) “*If we had a clean sanitary environment, we would not be at risk for polio, so why doesn’t the government focus on that?*” (Refusal Mother)
	Coercive enforcement approaches	“*Parents are told that if they do not give polio drops, the police will take them away or schools will be sealed*.” (Government Stakeholder)
**Communication and Operational Recommendations**	Role of trusted individuals	“*When people are guided by people they trust from the community whether it be their own tribe, leaders, doctors, neighbors or celebrities, refusal conversion is smoother*.” (Frontline Worker) “*A doctor only needs to say: ‘If your child gets polio, there is no treatment.’ That would have more impact than repeated pressure*.” (Government Stakeholder)
	Interdepartmental Coordination	“*Elected members, doctors, and clinic staff must all be more actively involved in counseling parents. Since they listen to people on high posts*.” (Government Stakeholder) “*Strengthen inter-department coordination (KMC, WASA); launch “Clean Lane Campaign” before polio round*.” (Government Stakeholder)
	Integrated Service Delivery	“*Every visit to a government dispensary or MCH center should include a dedicated polio verification desk to check a child’s vaccination status before consultation, followed by counselling and vaccination if the child is unvaccinated***.”** (Frontline Worker)
	Targeted awareness sessions for refusal parents	“*I would go if there were any awareness sessions being held and if doctors and healthcare professionals are leading these sessions*.” (Refusal Mother)
	Program strengthening	“*There should be educated and trained workers who can explain things logically and answer questions confidently*.” (Refusal Father) “*Effective monitoring and timely data (received by evening) make next-day planning clear. UC-level monitoring and ERMs should be strengthened*.” (Government Stakeholder)

## Data Availability

The data generated and analyzed during this study are not publicly available due to privacy and ethical restrictions, as the qualitative data may contain information that could potentially identify individual participants. De-identified data may be made available from the corresponding author upon reasonable request, subject to applicable ethical and privacy requirements.
